# Social Networking Sites Addiction and Materialism Among Chinese Adolescents: A Moderated Mediation Model Involving Depression and Need to Belong

**DOI:** 10.3389/fpsyg.2020.581274

**Published:** 2020-12-08

**Authors:** Pengcheng Wang, Li Lei, Guoliang Yu, Biao Li

**Affiliations:** ^1^School of Education, Renmin University of China, Beijing, China; ^2^Institute of Psychology, Renmin University of China, Beijing, China; ^3^School of Journalism and Communication, Renmin University of China, Beijing, China

**Keywords:** social networking site addiction, materialism, depression, need to belong, adolescent

## Abstract

Recent research indicates that social networking site (SNS) addiction is positively associated with materialism. However, little attention has been paid to the potential mechanisms in this relationship. This study tested the mediating role of depression and the moderating role of need to belong (NTB) in the relationships between SNS addiction and adolescents’ materialism. This research model was tested among 733 adolescents in China (mean age = 16.79 years, *SD* = 0.91). The findings indicated that both SNS addiction and NTB were positively related to adolescents’ materialism. Mediation analyses showed that depression mediated the association between SNS addiction and adolescents’ materialism. Moderated mediation indicated that the effect of SNS addiction on materialism and the effect of SNS addiction on depression were exacerbated by NTB. This study can advance our understanding of how SNS could contribute to adolescents’ materialism in this digital society.

## Introduction

Social networking sites (SNSs) have become irreplaceable in adolescents’ daily life (Kuss and Griffiths, 2011). SNS allow users to keep in touch with others *via* diverse forms, including sending messages, updating status, posting comments, and viewing the information posted by other users (Ellison et al., 2007), which are changing the ways that we communicate with each other. However, SNS users may experience symptoms of addictions that were traditionally related to substance use (Kuss and Griffiths, 2011). SNS addiction, also known as overuse of SNS or problematic SNS use, is garnering increasing academic attention given its adverse effects. For example, SNS addiction could undermine personal well-being, damages academic performance, and leads to interpersonal conflicts (Kuss and Griffiths, 2011; Wang et al., 2017, 2018c; Yin et al., 2019). Recent empirical research has also related SNS addiction to materialism (Sharif and Khanekharab, 2017).

Materialism involves a set of values and goals focused on possessions, wealth, image, and personal status (Kasser, 2016). Materialism is often defined as “the importance ascribed to the ownership and acquisition of material goods in achieving major life goals or desired states” (Richins and Dawson, 1992). Prior research has linked materialism to many negative personal situations including low life satisfaction, low relationships quality, more consumption problems, and poorer well-being (Dittmar et al., 2007, 2014; Wang et al., 2017). Additionally, there is an alarming concern that the younger generations are becoming more materialistic (Schaefer et al., 2004). Based on the cultivation theory, prior research indicated that media usage could increase consumption perceptions and lead heavy users to become materialistic (Sirgy et al., 1998). In line with this notion, ample evidence suggested that traditional media like TV could lead to materialism (Richins, 1987; Sirgy et al., 1998, 2012). Moreover, a recent study found that new media addiction like smartphone addiction was associated with adolescents’ materialism (Wang et al., 2018b). One underlying reason is that new media like smartphones has become important avenues for advertises (CNNIC, 2018). These advertises often contain materialistic information, which may facilitate the users’ materialistic values over time (Richins, 1987). Therefore, the addicted users are in greater chance to be exposed to the advertising information and become more materialistic (Wang et al., 2018b). Given that SNS has also become common avenues for advertising (CNNIC, 2018), it is likely that people high in SNS addiction may become more materialistic due to their exposures to these advertises. In addition, since the incidence rate of SNS addiction is relatively high (Kuss and Griffiths, 2011), it is vital to test the link between SNS addiction and materialism.

However, only one study has tested the link between SNS addiction and materialism (Sharif and Khanekharab, 2017), and the study only tested the direct relationship between SNS addiction and materialism, while the mediating (i.e., how SNS addiction is related to materialism) and moderating mechanisms (i.e., for whom is this relationship most potent) remain largely unknown. Meanwhile, this study only focused on young adults (Sharif and Khanekharab, 2017), much less is known regarding the link between SNS addiction and materialism among adolescents.

Thus, our study established a moderated mediation model to explore the mediating effect of depression and the moderating effect of need to belong (NTB) in the link between SNS addiction and materialism among a sample of Chinese adolescents.

### The Mediating Role of Depression

Prior research argues that Internet addiction reflects a form of passive coping, which means that the users often count on the Internet to elude their troubles or problems in the real world (Caplan, 2002; Wang et al., 2018c). The coping style theory suggests that passively coping with one’s problems may damage one’s personal well-being (Lazarus and Folkman, 1984). Given that SNS addiction is a subtype of Internet addiction, it is possible that SNS addiction could contribute to depression. Empirical data support this assumption. A cross-sectional study indicated that SNS addiction is positively associated with adolescent depression (Wang et al., 2018c). Additionally, diary and experience sampling research revealed that SNS use is a predictor of depression (Steers et al., 2014; Verduyn et al., 2015).

Depression may contribute to adolescents’ materialism. Prior research suggested that materialism can function as a form of coping strategy to compensate for people’s feelings of psychological insecurity (Dittmar et al., 2014). Put differently, people with a high level of psychological insecurity are more likely to have a higher level of materialism. Given that depression is positively and significantly related to psychological insecurity (Feeney et al., 2003; Fowler et al., 2013), it is likely that depression can predict adolescents’ materialism. Existing research supports this notion. A number of studies indicated that depression is positively linked to people’s materialism (Kashdan and Breen, 2007; Mueller et al., 2011; Ya Azibo, 2013). However, the mediating effect of depression in the relationship between SNS addiction and adolescents’ materialism has not been examined. Thus, the following hypothesis was established:

*Hypothesis* 1: SNS addiction will be positively associated with adolescent depression, which in turn will be positively associated with adolescents’ materialism. In other words, depression will mediate the link between SNS addiction and adolescents’ materialism.

### The Moderating Role of NTB

Although SNS addiction could impact adolescents’ materialism *via* depression, not all adolescents will be equally impacted by this effect. Thus, it would be beneficial to explore potential moderators in relationships between SNS addiction and adolescents’ materialism. Our study would test the hypothesis that NTB would moderate the relationships between SNS addiction and adolescents’ materialism.

The belongingness hypothesis suggests that NTB is a fundamental, powerful, and pervasive human motivation (Baumeister and Leary, 1995). It profoundly impacts individual cognitions, emotions, and behaviors. People high in NTB are more sensitive about their relations with other people (Baumeister and Leary, 1995). Thus, they are more likely to be high in relational insecurity and have high levels of unmet needs for relatedness. In addition, prior research indicated that relational insecurity led people to increasingly focus on materialistic strivings (Sheldon and Kasser, 2008; Dittmar et al., 2014). Besides, basic psychological need satisfaction, including relatedness, is negatively related to materialism (Kashdan and Breen, 2007; Tsang et al., 2014). Therefore, it is logical to deduce that NTB could be positively related to materialism. However, little research attention has been paid to the link between NTB and materialism.

NTB could moderate the effect of SNS addiction on adolescents’ materialism. Based on the compensatory Internet use hypothesis (Kardefelt-Winther, 2014), Internet addiction often derives from people’s unsatisfaction in real life. Given that the main functions of SNS are helping people to interact and connect with each other, as a specific form of Internet addiction; SNS addiction may reflect people’s unsatisfaction about their interpersonal relationships in real life. In other words, SNS addiction may be regarded as an indicator of negative interpersonal connections in the offline world. As the diathesis-stress framework suggests, negative personal traits may exacerbate the effects of adverse environmental factors on people (Monroe and Simons, 1991; Belsky and Pluess, 2009). Given that an extremely high level of NTB is associated with a series of negative outcomes, such as bullying and impaired mental health, sometimes, it can be regarded as a negative trait (Reichl et al., 2013; Underwood and Ehrenreich, 2014). Therefore, NTB may moderate the relationships between SNS addiction and adolescents’ materialism. Prior research showed that NTB could indeed moderate the effects of some factors on individual outcomes. For instance, a study showed that NTB could exacerbate the link between self-esteem and adolescents’ smartphone addiction (Wang et al., 2017b).

To date, little research has explored NTB as a predictor of adolescents’ materialism or as a moderator in the relationships between SNS addiction and adolescents’ materialism. Given these thoughts, the following hypotheses were built:

*Hypothesis* 2: NTB would moderate the links between SNS addiction and adolescents’ materialism *via* depression. Specifically, the relationships between SNS addiction and adolescents’ materialism would be stronger for adolescents high in NTB.

### The Present Study

**Figure 1:**
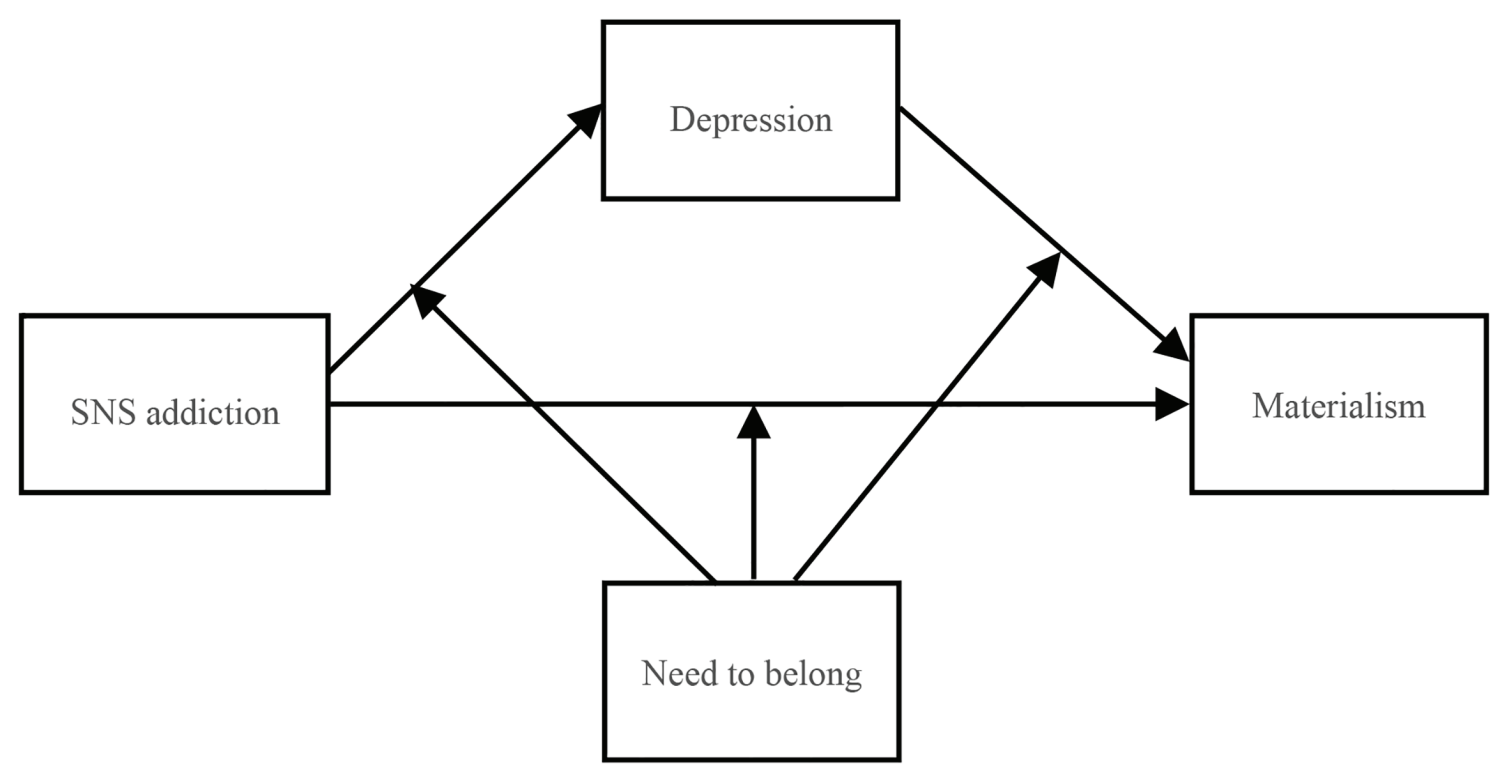
The proposed moderated mediation model.

In our study, a conceptual model of the process by which SNS addiction is positively related to adolescents’ materialism was tested. To be specific, the aim of the present study was 2-fold: to test whether depression would mediate the link between SNS addiction and adolescents’ materialism and to test whether NTB would moderate the links between SNS addiction and adolescents’ materialism *via* depression. These two research hypotheses make up a moderated mediation model. Figure 1 shows the proposed model.

## Materials and Methods

### Participants

The participants were from two senior high schools in Hebei Province, China. A total of 733 students participated in this study. Their average age was 16.70 years (*SD* = 0.91, range = 15–19 years; 43% males). Fifty-three percent of the participants were from grade 10 and the rest of them were from grade 11. The study variables involved in this study were part of our survey studies, including demographic information, SNS addiction, materialism, depression, and NTB.

### Measures

#### SNS Addiction

The Chinese version of the SNS Intrusion Questionnaire (CSIQ) was used to assess the participants’ SNS addiction. The CSIQ was adapted from the Facebook Intrusion Questionnaire (FIQ; Elphinston and Noller, 2011), and it has been used in the prior research among adolescents from China (e.g., Wang et al., 2018c; Yin et al., 2019). The CSIQ contains eight items (“I often use SNS for no particular reason”). Participants rated each item with on a 7-point scale (1 = *never*, 7 = *always*). Higher average scores indicate worse SNS addiction conditions. Cronbach’s α for the CSIQ was 0.87 in our study.

#### Materialism

Materialism was assessed by the Material Values Scale (MVS; Richins and Dawson, 1992). The MVS contains 18 items (e.g., “My life would be better if I owned certain things I do not have”), which can be divided into three subscales, including pursuit of happiness through acquisition, acquisition centrality, and possession-defined success. It has been used in previous studies among the Chinese participants and showed good psychometrics (Wang et al., 2018b). Participants rated all items on a 5-point scale (1 = *strongly disagree* to 5 = *strongly agree*), with higher average scores, indicating higher levels of materialism. Cronbach’s α for the MVS was 0.70 in our study.

#### Depression

Depression was assessed by the Chinese version of the Center for Epidemiological Studies Depression Scale (CES-D; Radloff, 1977). This scale contains 20 items (e.g., “I had crying spells”). It has been prevalently used among the Chinese adolescents (e.g., Wang et al., 2017a, 2018a,c). The participants indicated the frequency that they experienced the symptoms with the last week on a 4-point scale (0 = “rarely or none of the time”; 1 = “some or little of the time”; 2 = “occasionally or a moderate amount of time,” and 3 = “most or all of the time”), with higher average scores indicating higher levels of depression. Cronbach’s α for the CES-D was 0.93 in our study.

#### Need to Belong

NTB was assessed by the Single-Item Need to Belong Scale (SI-NTBS; Nichols and Webster, 2013), and this scale has been identified to have good reliability and validity in prior research (Nichols and Webster, 2013). The SI-NTBS has been widely adopted in prior research including the Chinese samples (e.g., Wang et al., 2017b). The item is “I have a strong need to belong.” The participants rated it on a 5-point scale (1 = *not at all*, 5 = *extremely*), with higher scores indicating higher levels of NTB.

### Procedure

The Research Ethics Committee of the corresponding author’s university approved this study. The data were collected in the participants’ classrooms from March 2017 to May 2017. Experienced psychology graduate students conducted the data collection activities. The informed consent was acquired from the students’ parents and their teachers. All participants were notified that their involvement was totally voluntary, and they can terminate their participation if they want. Each participant was given a small gift as incentives.

### Statistical Analyses

First, the descriptive information and correlation matrix were conducted. Second, we calculated the mediation analyses according to the four-step procedure (Baron and Kenny, 1986). Third, Hayes’s (2013) PROCESS macro (Model 59) was adopted to conduct the moderation mediation analyses. All continuous variables were standardized while conducting these analyses. Besides, we applied the bootstrapping approach to examine the significance of all the effects to gain robust standard errors for parameter estimation (Hayes, 2013). The 95% bias-corrected confidence intervals of these effects from 2000 resamples of the data were conducted. The data analyses were calculated with the IBM SPSS Statistics, Version 21.

## Results

### Examination of the Common Method Bias

Given that the measurements of the study variables were self-reported, we used the Harman’s one-factor test to examine the degree that the relationships among the study variables may come from common method variance. Common method bias would exist if one general factor or a single factor accounts for most of the variance (Podsakoff et al., 2012). Unrotated factor analysis showed that the first factor explained only 20.62% of the variance, which indicates that common method bias is not likely to be a severe issue in our study.

### Preliminary Analyses

The means, SD, and Pearson correlation coefficient of the study variables are shown in Table 1. As expected, SNS addiction was positively related to materialism, depression, and NTB. Moreover, depression and NTB were positively related to materialism. In addition, NTB was positively related to depression.

**Table 1:** Descriptive statistics and correlations between variables.

	*M*	*SD*	1	2	3	4
1. SNSA	3.67	1.10	1			
2. Mat	2.80	0.52	0.30^***^	1		
3. Dep	0.97	0.52	0.26^***^	0.21^***^	1	
4. NTB	3.44	0.94	0.14^***^	0.22^***^	0.20^***^	1

*N* = 733. SNSA, social networking sites addiction; Mat, Materialism; and Dep, Depression.

*** *p* < 0.001.

### Testing for Mediation

The four-step procedure was adopted to examine the mediation hypothesis (Baron and Kenny, 1986). This procedure demands (a) the relationship between SNS addiction and materialism to be significant; (b) the relationship between SNS addiction and depression to be significant; (c) the relationship between depression and materialism to be significant while controlling for SNS addiction; and (d) a significant effect for the indirect link between SNS addiction and adolescents’ materialism *via* depression. We used the bias-corrected percentile bootstrap method to determine whether the last condition is up to the standard.

As the multiple regression analyses showed (Table 2), in the first step, that SNS addiction was positively associated with adolescents’ materialism, *β* = 0.30, *p* < 0.001. In the second step, SNS addiction was positively associated with depression, *β* = 0.26, *p* < 0.001. In the third step, depression was significantly related to adolescents’ materialism while controlling for SNS addiction, *β* = 0.14, *p* < 0.001. At last, the bias-corrected percentile bootstrap method indicated that the indirect effect was significant, *ab* = 0.036, *SE* = 0.011, 95% *CI* = [0.016, 0.059]. Thus, the mediating effect was significant.

**Table 2:** Testing the mediation effect of social networking site (SNS) addiction on materialism.

Predictors	Model 1 (Mat)		Model 2 (Dep)		Model 3 (Mat)	
	*β*	*t*	*β*	*t*	*β*	*t*
SNSA	0.30	8.45^***^	0.26	7.38^***^	0.26	7.23^***^
Dep					0.14	3.77^***^
*R*^2^	0.09		0.07		0.11	
*F*	71.40^***^		54.40^***^		43.45^***^	

*N* = 733. Each column is a regression model that predicts the criterion at the top of the column. SNSA, social networking sites addiction; Mat, materialism; and Dep, depression.

*** *p* < 0.001.

### Testing for Moderated Mediation

As shown in Table 3 (Model 1), we tested the moderating role of NTB on the link between SNS addiction and depression. In Model 2, we tested the moderating effect of NTB on the link between SNS addiction and materialism, and the moderating effect of NTB on the link between depression and adolescents’ materialism.

**Table 3:** Testing the moderated mediation effect of SNS addiction on materialism.

Predictors	Model 1 (Dep)		Model 2 (Mat)	
	*β*	*t*	*β*	*t*
SNSA	0.23	6.55^***^	0.25	6.82^***^
NTB	0.17	4.70^***^	0.17	4.85^***^
SNSA × NTB	0.09	2.72^**^	0.07	2.02^*^
Dep			0.10	2.86^**^
Dep × NTB			−0.03	−1.05
*R*^2^	0.11		0.14	
*F*	28.54^***^		23.28^***^	

*N* = 733. SNSA, social networking sites addiction; Mat, materialism; and Dep depression.

* *p* < 0.05

** *p* < 0.01

*** *p* < 0.001.

**Figure 2:**
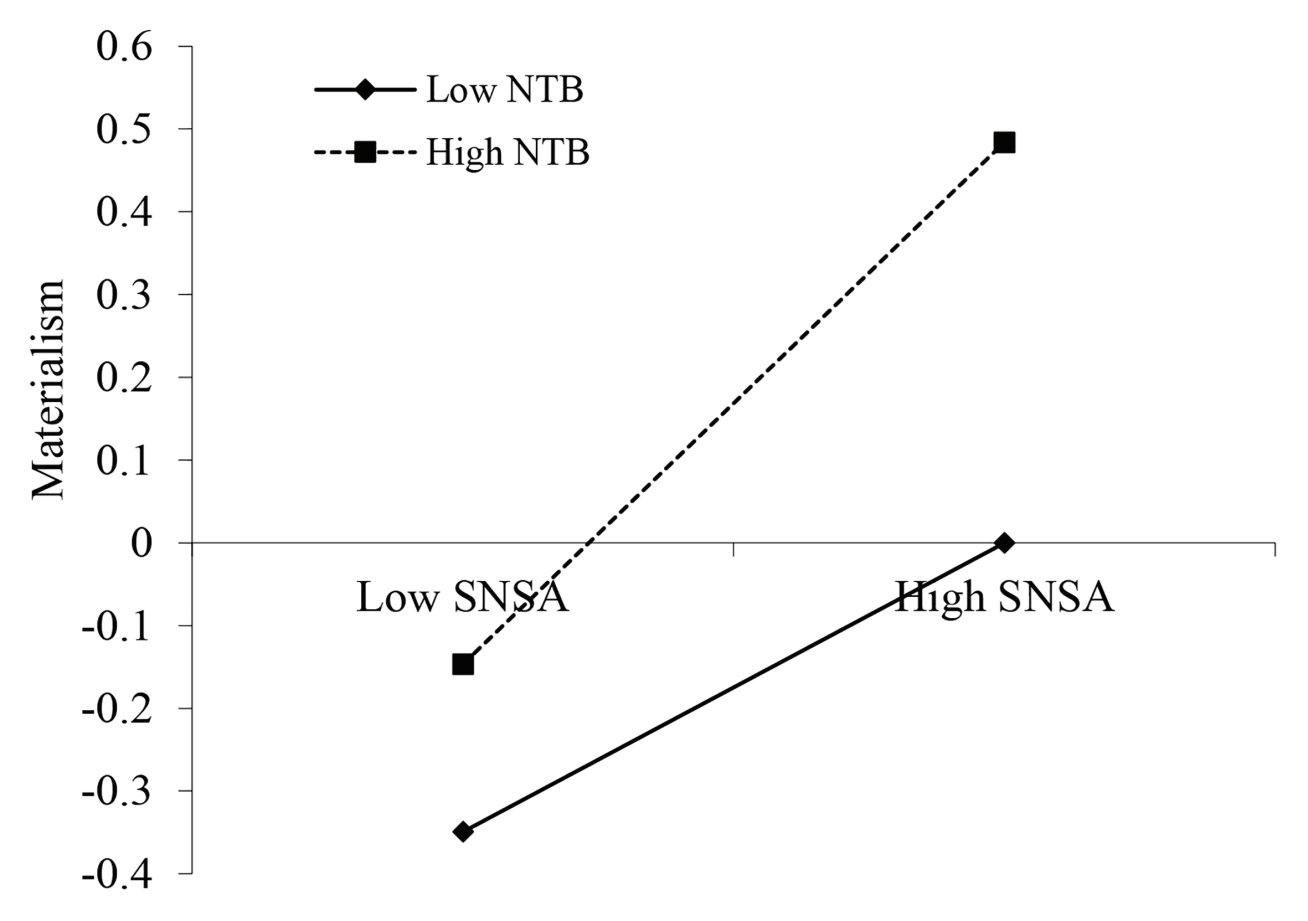
Need to belong (NTB) moderates the link between SNS addiction and materialism. SNSA, SNS addiction.

**Figure 3:**
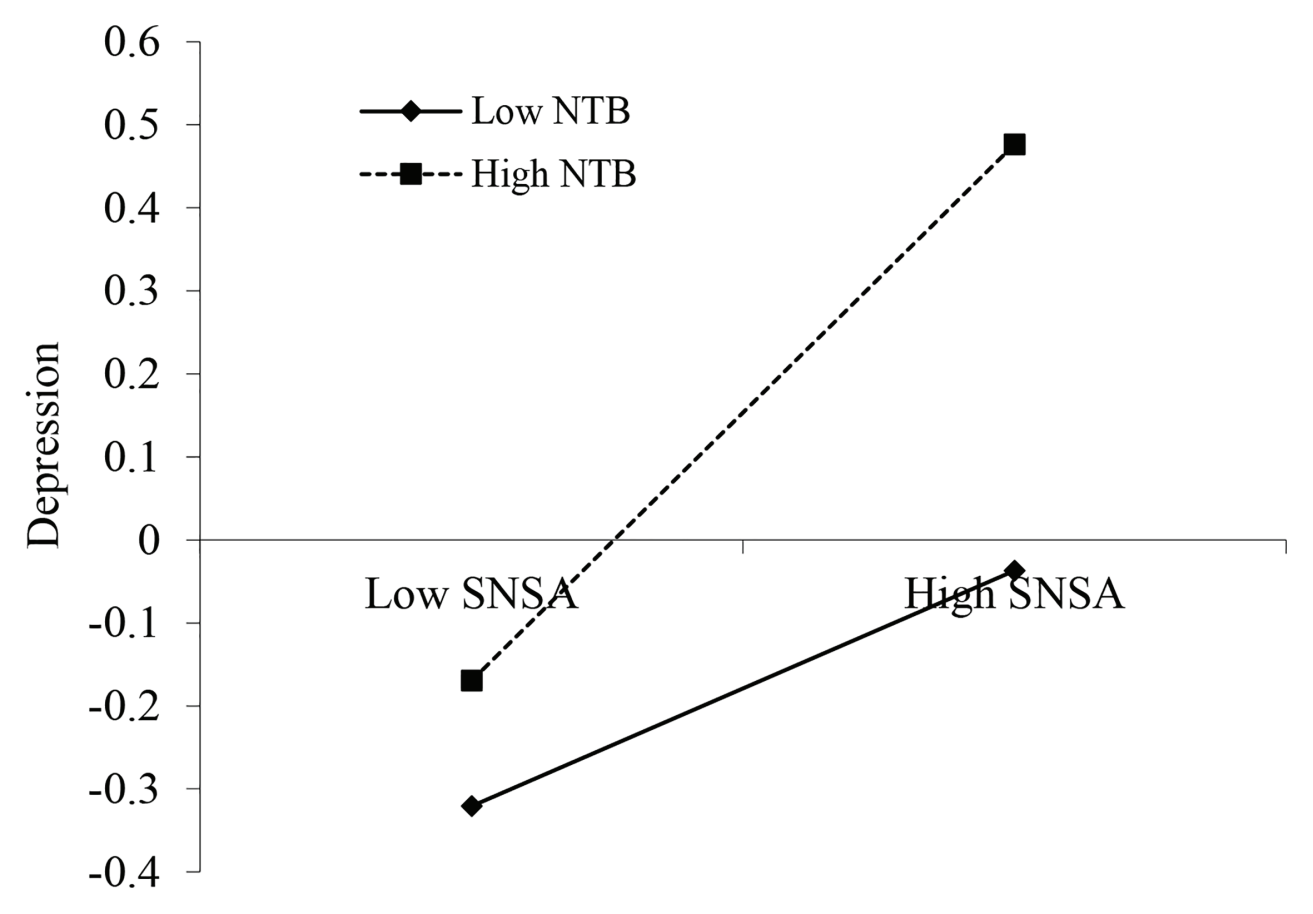
NTB moderates the link between SNS addiction and depression.

As Table 3 shows, there was a significant main effect of SNS addiction on adolescents’ materialism (*β* = 0.25, *p* < 0.001), and this effect was moderated by NTB (*β* = 0.07, *p* < 0.05). For clarity, we plotted the regression of adolescents’ materialism on SNS addiction at high and low levels of NTB (1 *SD* below the mean and 1 *SD* above the mean, respectively; Figure 2). The simple slope tests showed that, for adolescents high in NTB, high level of SNS addiction was related to high level of materialism (*β*_simple_ = 0.32, *p* < 0.001). For adolescents low in NTB, the link between SNS addiction and adolescents’ materialism was much weaker (*β*_simple_ = 0.18, *p* < 0.001). Model 1 of Table 3 showed that there was a significant effect of SNS addiction on depression (*β* = 0.23, *p* < 0.001), and this effect was moderated by NTB (*β* = 0.09, *p* < 0.01). For clarity, the regression of adolescent depression on SNS addiction at high and low levels of NTB (1 *SD* below the mean and 1 *SD* above the mean, respectively) was plotted (Figure 3). Simple slope tests showed that for adolescents high in NTB, high level of SNS addiction was associated with high level depression (*β*_simple_ = 0.32, *p* < 0.001). For adolescents low in NTB, the link between SNS addiction and adolescent depression was much weaker (*β*_simple_ = 0.14, *p* < 0.01). At last, Model 2 of Table 3 indicated that the main effect of depression on materialism was significant (*β* = 0.10, *p* < 0.01), and it was not moderated by NTB (*β* = −0.03, *p* > 0.05).

## Discussion

Recent research has started to examine the relationships between new media addiction (e.g., SNS/smartphone addiction) and people’s materialism (Sharif and Khanekharab, 2017; Wang et al., 2018b). However, the underlying mechanisms in the link between SNS addiction and adolescents’ materialism stay largely unknown. Our study set up a moderated mediation model to examine whether depression mediated the relationship between SNS addiction and adolescents’ materialism and whether NTB moderated the relationships between SNS addiction and adolescents’ materialism. The results showed that the influence of SNS addiction on adolescents’ materialism can be partially explained by depression. Moreover, the direct relationship between SNS addiction and adolescents’ materialism was moderated by NTB, and the indirect relationship was moderated by NTB in the first stage of the mediation process as well, with both effects in these two relationships being stronger for adolescents high in NTB. We will discuss each research hypotheses in the following sections.

### The Mediating Role of Depression

This study showed that SNS addiction was positively associated with adolescents’ materialism, and depression mediated this relationship. To be specific, SNS addiction was positively linked to depression, which in turn was positively related to materialism (i.e., SNS addiction → depression → materialism). Therefore, the depression could be one explanatory factor for how SNS addiction could contribute to adolescents’ materialism. To be specific, these findings suggest that SNS addiction can impair adolescents’ mental health, which in turn can lead them to adopt a high level of materialism to make up for their psychological insecurity (Dittmar et al., 2014). These findings are consistent with prior research showing that SNS addiction is positively associated with university students’ materialism (Sharif and Khanekharab, 2017). Given that SNS are becoming irreplaceable in many adolescents’ lives, they could be the additional media that can lead adolescents to become materialistic apart from traditional media like TV, and these findings can provide a more comprehensive understanding of why adolescents are becoming more materialistic (Schaefer et al., 2004; Kasser, 2016). This study goes beyond previous research by illuminating how new media like SNS addiction could encourage adolescents’ materialism, which can be of great help in the prevention of and interventions for adolescents’ materialism in this digital era.

Beyond the mediating effect, the separate relationships in this mediation model are also noteworthy. In the first stage of mediation (i.e., SNS addiction → depression), our findings support recent research showing that SNS addiction is positively linked to adolescents’ depression (Wang et al., 2018c). This finding also supports the idea that as a form of passive coping (Caplan, 2002; Wang et al., 2018c), and Internet addiction, including SNS addiction, can undermine adolescent well-being. In the second stage of mediation (i.e., depression → materialism), our study found that depression was positively linked to adolescents’ materialism. This finding is congruent with existing research showing that people high in depression are at more odds of having a higher level of materialism (Kashdan and Breen, 2007; Mueller et al., 2011; Ya Azibo, 2013). This finding is consistent with the notion suggesting that materialistic strivings can be a way of coping with feelings of psychological insecurity (Dittmar et al., 2014).

### The Moderating Role of NTB

Another goal of our study was to find out whether NTB would be positively related to adolescents’ materialism and whether NTB could moderate the relationships between SNS addiction and adolescents’ materialism. The results found that NTB was positively related to adolescents’ materialism. Our study is the first to document such finding in this literature. Considering that adolescents high in NTB are more likely to be unsatisfied with their current relationships, they have more risk of having unmet basic psychological needs, such as relatedness (Baumeister and Leary, 1995). Our finding supports the notion that unmet basic psychological needs like relatedness can positively predict materialism (Kashdan and Breen, 2007; Tsang et al., 2014).

Moreover, this study found that NTB moderated the direct link between SNS addiction and adolescents’ materialism. Specially, the relationship between SNS addiction and materialism was stronger for adolescents with high levels of NTB. As the belongingness hypothesis suggests (Baumeister and Leary, 1995), adolescents high in NTB often make more effort to seek social connections and fit in with their groups. Therefore, they are more likely to accept social norms preached by business institutions on new media, including SNS; because these norms usually contain materialistic messages or values, it makes sense that the relationship between SNS addiction and adolescents’ materialism was stronger among adolescents high in NTB. Thus, NTB could be treated as a helpful index to distinguish whether people addicted to new media, including SNS, are at more risk of having materialistic values.

In addition, this study found that NTB moderated the relationship between SNS addiction and adolescents’ depression (first stage moderation). In other words, the relationship between SNS addiction and depression was stronger for adolescents high in NTB. As previous research showed, new media use could undermine users’ relationships with others (Muise et al., 2009; Morry et al., 2018). Considering that people high in NTB are more sensitive about their social relationships (Baumeister and Leary, 1995), it is logic to find that the link between SNS addiction and depression was stronger among adolescents high in NTB.

Unlike we assumed, NTB did not moderate the association between depression and adolescents’ materialism. One possible explanation is that depression has a powerful influence on adolescents’ materialism (Kashdan and Breen, 2007; Mueller et al., 2011; Ya Azibo, 2013). Therefore, adolescents who are highly depressed will be highly materialistic regardless of their level of NTB.

### Implications

Our study has several important theoretical implications. Our study shows that SNS addiction is positively linked to adolescents’ materialism, which can provide us with a more comprehensive understanding of why adolescents are becoming more materialistic in this digital society. Although prior research has found that traditional media (e.g., TV) can encourage materialistic values over the long-term by spreading materialistic messages like advertises (Richins, 1987; Sirgy et al., 1998, 2012), research has just begun to examine the link between new media and materialism (Sharif and Khanekharab, 2017; Wang et al., 2018b). Our study showed that SNS addiction is positively associated with adolescents’ materialism. Besides, depression mediates the association between SNS addiction and adolescents’ materialism. NTB exacerbates the association between SNS addiction and depression as well as the association between SNS addiction and materialism. Given that SNS have become indispensable in many adolescents’ lives and given the negative influences of materialism on adolescents’ development (Dittmar et al., 2014), all these findings are beneficial for us to understand how SNS addiction could be linked to adolescents’ materialism.

In addition, our study has some practical implications. First, this study indicates that it is vital for policymakers, parents, and school educators to attempt to reduce adolescents’ dependence on new media, including SNS. Second, our study can help to people understand how SNS addiction is related to adolescents’ materialism, which can shed light on prevention and intervention efforts. For example, prevention and intervention efforts aimed at reducing depression (e.g., mindfulness exercises) can decrease materialism among adolescents (Friis et al., 2016). Third, although SNS addiction is positively linked to depression and materialism among adolescents, the effects differed for adolescents with diverse levels of NTB. We should prioritize prevention and intervention for adolescents high in NTB.

### Limitations and Future Directions

Some limitations should be reminded. First, our study was cross-sectional designed and, therefore, causality cannot be confirmed. Researchers could use experimental or longitudinal data to confirm the causal assumptions. Second, the measures in our study were self-reported, so future researchers can collect data from diverse informants to test these hypotheses. Third, this study only used one item to assess participants’ NTB; although this measure has been proven valid and been widely used in prior studies, future studies can use a more sensitive measurement like the 10-item the NTB Scale to examine the relationship found in the present study (Leary et al., 2013; Wang et al., 2018d). Finally, our study examined a sample of students instead of a clinical sample, we should be careful when generalizing these results to other samples. It would be helpful for future researchers to examine this model in people from other groups, such as the clinical patients.

## Conclusion

In summary, our study shows that SNS addiction is positively linked to adolescents’ materialism. Additionally, mediation analysis indicated that depression mediates the association between SNS addiction and adolescents’ materialism. Furthermore, NTB moderated the association between SNS addiction and materialism, as well as the association between SNS addiction and depression, with both effects being stronger for adolescents high in NTB.

## Data Availability Statement

The raw data supporting the conclusions of this article will be made available by the authors, without undue reservation.

## Ethics Statement

The studies involving human participants were reviewed and approved by the Research Ethics Committee of Renmin University of China. Written informed consent to participate in this study was provided by the participants’ legal guardian/next of kin.

## Author Contributions

PW: conceptualization, software, and writing – original draft. LL: review and editing. GY: review and editing. BL: supervision and validation. All authors contributed to the article and approved the submitted version.

### Conflict of Interest

The authors declare that the research was conducted in the absence of any commercial or financial relationships that could be construed as a potential conflict of interest.
